# Human Exposure to Ferret Badger Rabies in Taiwan

**DOI:** 10.3390/ijerph15071347

**Published:** 2018-06-27

**Authors:** Tai-Hwa Shih, Jeng-Tung Chiang, Hung-Yi Wu, Satoshi Inoue, Cheng-Ta Tsai, Shih-Chiang Kuo, Cheng-Yao Yang, Chang-Young Fei

**Affiliations:** 1 Bureau of Animal and Plant Health Inspection and Quarantine, Taipei 10070, Taiwan; hcshih@mail.baphiq.gov.tw (T.-H.S.); tctaga@mail.baphiq.gov.tw (C.-T.T.); kuosc@mail.baphiq.gov.tw (S.-C.K.); 2 Department of Statistics, National Chengchi University, Taipei 11605, Taiwan; chiangj@nccu.edu.tw; 3 Graduate Institute of Veterinary Pathobiology, College of Veterinary Medicine, National Chung-Hsing University, Taichung 40227, Taiwan; hwu2@dragon.nchu.edu.tw; 4 National Institute of Infectious Disease, Tokyo 162-8640, Japan; sinoue@nih.go.jp; 5 Agricultural Technology Research Institute, Hsinchu 30093, Taiwan; cyyang@mail.atri.org.tw; 6 School of Veterinary Medicine, National Taiwan University, Taipei 10617, Taiwan

**Keywords:** ferret badger, *Melogale moschata*, Taiwan, rabies, human exposure

## Abstract

On 17 July 2013, Taiwan confirmed multiple cases of the rabies virus (RABV) in the wild Taiwan Ferret badger (TFB) (*Melogale moschata*) member of the family *Mustelidae*. This study aims at investigating the risk factors for human exposure to rabid TFBs. Statistical inference based on Pearson correlation showed that there was a strong positive correlation between the total number of positive TFB rabies cases and the number of rabid TFBs involved with human activities in 81 enzootic townships (*r* = 0.91; *p* < 0.001). A logistic regression analysis indicated that the risk probability of a human being bitten by rabid TFBs was significantly higher when there were no dogs around (35.55% versus 6.17% (indoors, *n* = 171, *p* = 0.0001), and 52.00% versus 5.26% (outdoors, *n* = 44, *p* = 0.021)), and whether or not there was a dog around was the only crucial covariate that was statistically significantly related to the risk of a human being bitten. In conclusion, this study showed the value of having vaccinated pets as a deterrent to TFB encounters and as a buffer to prevent human exposure to rabid TFBs. The presence of unvaccinated pets could become a significant risk factor in the longer term if rabies isn’t controlled in TFBs because of the spillover between the sylvatic and urban cycles of rabies. Consequently, raising dogs, as well as keeping rabies vaccinations up-to-date for them, can be considered an effective preventive strategy to reduce the risk for human exposure to rabid TFBs.

## 1. Introduction

On 17 July 2013, Taiwan confirmed multiple cases of the rabies virus (RABV) in wild Taiwan Ferret badgers (TFBs) [1,2], which marked the first appearance of sylvatic rabies in Taiwan since WHO declared Taiwan rabies-free in 1961 [3]. By December 2017, RABV has been detected in eight cases of spillover infection, comprising six Masked palm civets, one puppy (*Canis lupus familiaris*), and one house shrew (*Suncus murinus*), providing that the TFB is the sole reservoir species. Genetically, this TFB rabies virus variant is distinct from all other known rabies virus variants and can be categorized into two groups, i.e., the Phylogeny of nucleoprotein and glycoprotein genes from 59 Formosan FB-associated RABV revealed them to be clustered in two distinct groups, TW-I and TW-II groups, which are distributed in East and West Enzootic Areas, respectively, and was geographically separated by The Central Mountain Range [4,5,6]. These enzootic and non-enzootic areas in Taiwan island are geographically separated from the northern one-third of Taiwan by the Daan River and Heping River [5,7]. Up to April 2018, no rabid animals have been detected north of the two rivers, and no human rabies deaths have been associated with the TFB virus enzootic in Taiwan. Ever since 1999, the Bureau of Animal and Plant Health Inspection and Quarantine (BAPHIQ) has instituted passive and active surveillance programs for the detection of rabies in pets (dogs/cats), bats, and wild mesocarnivores, as well as other wildlife including the TFB, Masked palm civet (*Paguma larvata*), Small Indian civet (*Viverricula indica*), Crab-eating Mongoose (*Herpestes urva*), Siberian weasel (*Mustela sibirica davidiana*), Ferret (*Mustela putorius furo*), and Leopard cat (*Prionailurus bengalensis*), etc. [4,5]. A Chi-square test as well as a multivariable logistic regression analysis of 59 townships in the East endemic area of Taiwan revealed that townships elevated between 201 and 600 m and with large scale of forest, have a high probability for the incidence of TFB rabies [7,8]. Townships, being the smallest administrative units in Taiwan, which coincide with the boundaries of villages, cities, towns, or even districts, provide territorial and geographic information in Taiwan. Several townships commonly exist within a county or metropolitan city. The enzootic area in Taiwan island comprises three metropolitan cities (Taichung, Tainan, Kaohsiung) and 6 counties (Hualien, Taitung, Pingtung, Nantou, Yunlin, Chiayi) with a total of 217 townships. Among the 217 townships, 81 had positive TFB rabies cases, 133 had no cases, and 3 offshore island townships had no cases and were not included in this study. Current epidemiological data indicates that the temporal dynamics of TFB rabies is still sequestered to the above-mentioned enzootic area [5,7,8]. Humans are at risk mainly via exposure to the virus from dogs and cats that have been infected by rabid ferret-badgers. As part of a large-scale government response, mass vaccination of dogs and cats was conducted to provide an immunological barrier ever since 2013 [5] and to date this process has been ongoing. However, the prevalence and endemic range of rabies in the TFB still increases, and a total of 51 humans were bitten by rabid TFBs until April 2018. It is necessary to examine the potential factors accounting for the risk factors for human exposure to rabid TFBs. 

## 2. Materials and Methods

The area in this study comprised the East and West enzootic areas of TFB rabies in Taiwan island. All surveillance data concerning TFB rabies in this study were downloaded from the governmental website of BAPHIQ (http://www.baphiq.gov.tw/view.php?catid=10980). Sample collection and diagnostic examination for TFB rabies virus have been previously published [7]. Every case was geocoded by the location of townships. Once geocoded, rabies cases were assigned to the corresponding townships and the number of cases by township was categorized and calculated. Detailed account of every case recorded in the rabies incident reports submitted by local government veterinary services were kindly provided by BAPHIQ. 

MedCalc Statistical Software version 18.5 (MedCalc Software bvba, Ostend, Belgium; https://www.medcalc.org; 2018) was used to perform the data analysis. In logistic regression study, the number of cases of human exposure to rabid TFBs was defined as dependent variable (outcome); the number of cases whether dogs were present or not was defined as independent variable. Data analysis showed that the location of exposure (indoors or outdoors) did not contribute to the prediction of the outcome and this independent variable was removed from the model. The risk of human exposure to rabid TFBs as well as the corresponding relative risk, odds ratio and confidence intervals were calculated by comparing the number of the following two groups: (1) for indoors, the number of people being bitten or not being bitten by TFBs versus when dogs were present or not present; (2) for outdoors, the number of people being bitten or not being bitten by TFBs versus when dogs were present or not present. In the study of examining the relative risk and odd ratio, the “exposed group” were defined as “dogs were not present”. As for “human exposure”, it always indicates that the human has been exposed (bitten) by a rabid ferret badger, either indoors or outdoors. Non-rabid TFB attacks were not included in this study, since a rabid TFB strikes unprovoked attacks, which exhibits distinct mechanisms from those of non-rabid ones. 

## 3. Results

### 3.1. Numerical Correlation Estimation

**Figure 1:**
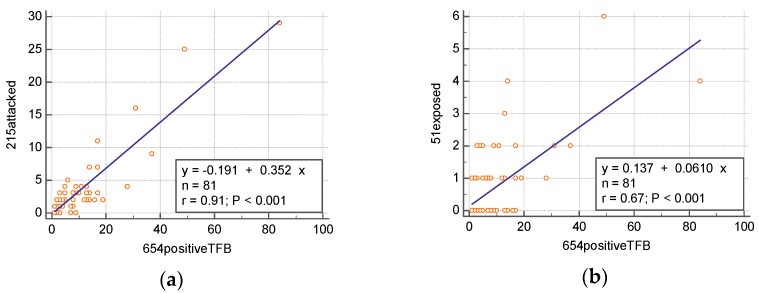
(**a**) Scatter diagram and regression line for regression of the number of 654 positive Taiwan Ferret badger rabies on the number of 215 rabid TFBs involved with human activities, *r* = 0.91. (**b**) Scatter diagram and regression line for regression of the number of 654 positive TFB rabies on the number of 51 cases exposed to rabid TFBs, *r* = 0.67. Data were collected from March 2010 to April 2018.

From March 2010 to April 2018, a total of 215 out of 654 rabies positive TFBs were found involved with human activities of daily lives. Among the 215 cases, there were 51 human exposure (bitten) to rabid TFBs. Figure 1a indicated that there was a high correlation between the number of positive TFB rabies and the number of rabid TFBs involved with human activities in 81 townships: *r* = 0.91; 95% confidence interval for the slope parameter was (0.3165–0.3894); *p* < 0.001. Figure 1b showed that the number of positive TFB rabies was moderately associated with the number of humans bitten by rabid TFBs in 81 townships: *r* = 0.67; 95% confidence interval for the slope parameter was (0.0460–0.0760); *p* < 0.001. Additionally, people living in hilly residential areas in Taiwan usually raise guard dogs. 

### 3.2. Logistic Regression Analysis 

Table 1 indicates the human exposure rate was related to the presence of dogs, logistic regression model was estimated as follows:

logit(p)=−0.44−2.31dog

The model fitted the data adequately and indicated that the human exposure was statistically associated with the presence of dogs (*p* < 0.0001).

### 3.3. The Risk Probability of Human Exposure to Rabid TFB

Table 2 indicated that among the 215 rabid TFBs involved with human activities, 51 (32 + 5 + 13 + 1) rabid TFBs bit humans. Of them, 37 (32 + 5) were found inside a house (indoors) and 14 (13 + 1) were found outside a house (outdoors). Logistic regression analysis found that whether there was a dog around was the only covariate that was statistically significantly related to the risk of human exposure to ferret badger rabies. Table 2 revealed that no matter rabid TFBs were found inside or outside a house, the risk probability of the human exposure to TFB rabies was statistically significantly higher when there was no dog around: 35.55% versus 6.17% (*p* = 0.0001) for indoors, and 52.00% versus 5.26% (*p* = 0.0210) for outdoors.

### 3.4. The Mantel-Haenszel Test

Table 3 presented the corresponding relative risks (RR), odds ratios (OR), and 95% confidence intervals (CI) comparing the risks of human exposure to rabid TFBs when dogs were not around against when there were dogs around. In indoor incidents, the risk of being bitten was 5.7 times higher and the odds was 8.4 times higher when dogs were not around. Under outdoor circumstances, the risk of being bitten was 9.8 times higher and the odds was 19.5 times higher when dogs were not around. Although the benefit of having a dog around seemed higher for an outdoor encounter, the Breslow-Day test failed to detect statistically significant differences between indoor and outdoor encounters (*p* = 0.481). Hence, the Mantel-Haenszel estimates for the common relative risk and the common odds ratio were also calculated and displayed in the bottom portions of Table 3. Overall, the risk of human exposure to a rabid TFB was about 6.5 times higher and the odds was 9.9 times higher when dogs were not around. The corresponding 95% confidence interval was (2.88, 14.63) for the relative risk, and (4.02, 24.51) for the odds ratio.

## 4. Discussion

This study investigated the risk of human exposure to TFBs. Logistic regression, Pearson correlation, and Mantel-Haenszel test were used to study causes of the risk of human exposure to TFBs. The total number of positive TFB rabies cases, total number of cases of rabid TFBs involved with human activities, and the total number of cases of human exposure (bitten) to rabid TFBs were used to perform the statistical analysis.

The epidemiology of canine rabies in the United States indicated that when the reservoir rabies was eliminated, the spillover infection (cattle) declined in a similar manner to that of the reservoir (dog) rabies. Similarly, a large number of stray and unvaccinated cats has contributed greatly to the cause of several large-scale exposures to humans [9]. The high correlation (*r* = 0.91) between the number of positive TFB rabies and the number of rabid TFBs involved with human activities, as shown in Figure 1, was similar with the above phenomenon. 

Shih et al. [7] pointed out that the size of forests was extremely associated with the incidence of ferret badger rabies (*p* < 0.001). Shih et al. [8] further pointed out that “Controlling for the altitude, higher positive ferret badger rabies cases were found associated with larger forest areas. On the other hand, when the forest area was controlled, higher positive ferret badger rabies cases were associated with lower altitude”. Ethologically, mustelids, despite their flexible food habits and social organization, are considered susceptible to urban development [10,11] due to their vulnerability to disturbance and the specific habitat requirements of setts, which accounts for the fact that the Taiwan Ferret badger, a member of Mustelidae, is not synanthropic and few rabid cases of them were found in urban areas [7,8,12]. Those facts also point out that a forest is a terrain prone to rabid TFBs. With the same altitude, the bigger forest a township encompassed, the larger area it had to accommodate TFBs. As the population of TFBs rises, so does the contact rate, and thus the frequent biting events, as shown in Figure 1.

Table 2 shows that there were 81 cases in which rabid TFBs trespassed indoors where dogs were kept by the owners. In 76 of the cases, the dog discovered the rabid TFBs before it attacked. The dog hence brawled with the TFBs to keep their owners from being bitten. The other 5 (81 − 76 = 5) cases of exposure occurred when the dogs were not on the spot of attacking. By the time the dog came to scuffle with the TFBs, their owners had been bitten. Also shown in Table 2 were 90 cases in which TFBs intruded indoors, where the owners did not keep dogs. In 58 of the cases, the attacking TFBs were spotted and killed by beating, whereas in 32 (90 − 58 = 32) of them, the TFBs were not killed until humans were bitten. This demonstrates that dogs were far more vigilant against territory intrusion and always rushed to expel transgressing TFBs. In those mountainous areas, guard dogs are usually kept as pets. Weighing above 20 kg, they usually have the physical advantage in repelling the trespassing TFBs. Also worth noting, is that all the capture records described the TFBs as “killed by both the owner and the dog before it was turned in to local veterinarian services.” It can be deduced that, when a rabid TFB intruded, the owner and the dog both put down the threat as if “they were both part of a pack”. As Beck and Katcher [13] indicated, from the human’s point of view, the dog is a member of the family, and from the dog’s perspective, the family is his/her pack. Borchelt et al. [14] also maintained that when an alien intrudes into a dog’s territory, it is followed by an attack of the whole pack. As for outdoor occasions, the dogs recorded in Table 2, which appeared in public areas, were mostly community dogs, or free-roaming dogs. In cases where rabid TFBs appeared in a public area, they usually showed up abruptly, followed shortly by sudden attacks to humans in the vicinity. Among the 19 recorded outdoor events where there were community or roaming dogs present, only 1 person was found bitten by TFBs. In the other 18 events, the rabid TFBs were invariably bitten to death by community dogs before they could attack. In the 25 cases recorded where there was no community or roaming dogs, by contrast, only 12 people were not bitten, leaving the other 13 cases, where humans fell victim to rabid TBFs’ attacks. As can be seen from the above, the community or free-roaming dogs inhabiting the natural areas or public areas can pose a significant threat to rabid TFBs. Therefore, the existence of community dogs should be considered of some value.

A meta-analysis of MedCalc version 18.5 uses the *Mantel*-*Haenszel* method for calculating the weighted pooled relative risk and odds ratio under the fixed effects model. In Table 3, the relative risk, odds ratio, and 95% confidence intervals further attest to the benefit of being accompanied by a dog so as not to be bitten (exposed) by a rabid TFB. All in all, the benefit of not being bitten by a rabid TFB when accompanied by a dog was apparent. 

Ever since the TFB rabies was confirmed in 2013, active surveillance programs have been consistently implemented to detect rabies in found-dead terrestrial mammals. These activities included roadside surveillance and active submission of found-dead ferret badgers. In an effort to ensure that the TFB virus had not shifted back into the Taiwan dog population, surveillance programs were instituted to identify and test rabies suspect dogs since 2013. Fluorescent antibody testing of brain tissue was used to confirm rabies infection [5]. The annual numbers of rabid animals, and the number of wildlife, as well as domestic pets to be tested, will be published (in progress). Concerning prevention programs, two health promotion campaigns were developed to improve public awareness of rabies and the importance of domestic pet and human vaccination. The first was the “2 don’t & 1 do” program, which includes the following instructions: do not abandon pets; do not contact wild animals; and do vaccinate your pets. The second was the “R-W-S-O principle”, which includes the following: remember what animal bit you (to tell doctor); wash with plenty of water; send patients to the hospital; and observe animals in quarantine for 10 days. Taiwan Centers for Disease Control (CDC), BAPHIQ, and the School of Veterinary Medicine, National Taiwan University created rabies websites to provide epidemic notifications as well as resources for extensive education. The vaccination rate has reached 97% of dogs and cats in enzootic villages at Taiwan [5]. Additionally, many health programs for rabies prevention have been ongoing during the time since 2013, comprising the following services: providing rabies-related information through annual seminars and workshops; holding advocacy campaigns in all levels of schools through the Ministry of Education; registering and vaccinating every dog adopted; and the Forestry Bureau, Council of Agriculture (COA) and Ministry of Interior campaign takes on the role of reminding people to avoid bringing pets into forest recreation areas and national parks through public announcements. 

Taiwan would like to one-day be re-classified as a rabies free area. Wildlife rabies reservoirs have been controlled and the virus eliminated from the reservoir species through intensive programs in Europe and North America. Currently BAPHIQ and Animal Health Research Institute (AHRI) are evaluating the potential success of programs of oral rabies vaccination of TFBs through aerial distribution vaccine in Taiwan in 2018. 

## 5. Conclusions

This study showed the value of having vaccinated pets as a deterrent to TFB encounters and as a buffer to prevent humans exposure to rabid TFBs. The presence of unvaccinated pets could become a significant risk factor in the longer term if rabies isn’t controlled in TFBs because of the spillover between the sylvatic and urban cycles of rabies. Consequently, raising dogs, as well as keeping rabies vaccinations up-to-date for them can be considered an effective preventive strategy to reduce the risk for human exposure to rabid TFBs.

## Figures and Tables

**Table 1:** Coefficients and Standard Errors in estimated logistic regression model of the human exposure rate on the presence of dogs.

Variable	Coefficient	Std. Error	Wald	*p*-Value
Presence of dogs	−2.30970	0.46240	24.9504	<0.0001
Constant	−0.44183	0.19107	5.3472	0.0208

**Table 2:** The risk probability of human bitten by rabid TFB, *n* = 215.

Entered Houses	Dog Present	Bitten by TFBs	Not Bitten by TFBs	Total	% Bitten Cases	*p*-Value
Yes	No	32	58	90	35.55	0.0001
(indoors ^1^)	Yes	5	76	81	6.17	
No	No	13	12	25	52.00	0.0210
(outdoors)	Yes	1	18	19	5.26	

^1^ indoors = rabid TFB entered human dwellings, and vice versa.

**Table 3:** The Relative risk, odds ratio, and 95% confidence interval comparing the situations without dogs around to those with dogs around, *n* = 215.

Association	Location	Relative Risk (95% CI)	Odds Ratio (95% CI)
Conditional	Indoors ^1^	5.7600 (2.357 14.075)	8.3862 (3.077 22.855)
	Outdoors	9.8800 (1.413 69.066)	19.5000 (2.246 169.285)
Common	-	6.492 (2.880 14.633)	9.926 (4.020 24.506)

^1^ indoors = rabid TFB entered human dwellings, and vice versa.
